# Active predators do not necessarily specialize in sedentary prey: A simulation model

**DOI:** 10.1111/nyas.15379

**Published:** 2025-05-21

**Authors:** Inon Scharf

**Affiliations:** ^1^ School of Zoology, The George S. Wise Faculty of Life Sciences Tel Aviv University Tel Aviv Israel

**Keywords:** area‐restricted search, competition, foraging mode, habitat use, movement ecology, predator–prey interactions

## Abstract

Predators employ diverse foraging modes, ranging from ambush to active pursuit of prey. While ambush predators are associated with capturing mobile prey, the specialization of active predators on sedentary prey remains less understood. I examined the circumstances under which active predators preferentially capture sedentary prey. Using a spatially explicit individual‐based simulation model, I manipulated the spatial patterns of sedentary prey, movement directionality, speed of mobile prey and active predators, and the presence of competing ambush predators. Key factors such as area‐restricted search (ARS) by active predators, uncertain capture success of prey, and prey reappearance after capture were also considered. The results suggest that active predators do not necessarily specialize in sedentary prey. Instead, their prey preference is influenced by prey spatial patterns and competition with ambush predators: clumped spatial patterns of sedentary prey and the use of ARS by active predators as well as competition with ambush predators drove active predators to focus on sedentary prey. Conversely, nondirectional movement by predators and faster‐moving prey often led to higher proportions of mobile prey being captured. These findings challenge traditional assumptions about active predator specialization and emphasize the importance of integrating spatial and behavioral dynamics into predator–prey models.

## Graphical abstract

Active predators, which move to find food, are assumed to specialize in sedentary prey. This study used an individual‐based simulation to test whether this assumption holds. The results show that active predators do not always specialize in sedentary prey; instead, prey choice greatly depends on prey spatial pattern and competition with ambush predators. This challenges traditional views, emphasizing the complexity of predator–prey interactions and the importance of considering spatial patterns.

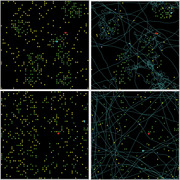

## INTRODUCTION

Animals in nature employ various strategies to locate prey, with active and ambush foraging modes representing opposite ends of a continuum.^1^, ^2^, ^3^ Intermediate foraging modes also exist. For instance, saltatory search combines movement phases—allowing for extensive area coverage—with stationary phases for prey search.^4^, ^5^ Sit‐and‐pursue predators ambush prey but may pursue it when detected.^6^ Pioneering studies since the 80s have shaped the development of the foraging mode paradigm.^7^, ^8^, ^9^, ^10^ While early works focused on lizards, the paradigm has since been applied to various taxa, including spiders, anurans, birds, snakes, and fish species.^9^, ^11^, ^12^, ^13^ Many factors, including prey, predator, habitat, and climate characteristics, can influence foraging mode selection.^14^, ^15^, ^16^ Active predators encounter prey more frequently than ambush predators, which benefit from energy conservation and reduced predation risk, enhancing their success despite less frequent encounters.^17^, ^18^, ^19^

Pianka^20^ originally distinguished two foraging modes: sit‐and‐wait and widely foraging predators. However, the concept of foraging modes was formally developed by the pioneering paper of Huey and Pianka,^8^ who proposed 11 key differences between the two foraging modes in terms of physiology, behavior, morphology, and life history. Several key differences have since been examined in greater depth through experimental and comparative studies. For example, widely foraging predators have higher metabolic rates, more elongated body shapes, and superior learning abilities than their sit‐and‐wait predator relatives.^19^, ^21^, ^22^ Other proposed differences have mixed support. For instance, ambush predators were suggested to have larger relative clutch masses, but some studies found no difference based on foraging mode or even the opposite.^23^, ^24^, ^25^

The first key difference in Huey and Pianka's paper^8^ between the two foraging modes is the type of prey captured, proposing that whereas ambush predators should focus on mobile prey, active predators should consume mostly sedentary prey. While it is clear why ambush predators can only capture active prey and not sedentary prey, it is less obvious why active predators would focus on sedentary prey. Few empirical studies have explored this preference. For example, Greene^26^ suggests that sedentary prey are easier to capture, more vulnerable, and less likely to escape compared to mobile prey. Indeed, two active predator species of marine copepods prefer early developmental stages of prey, which exhibit lower swimming speeds and reduced mobility compared to later stages.^26^ Additionally, predatory stoneflies more easily capture sedentary fly larvae than mobile mayflies probably because sedentary prey are less adept at evading active predators.^27^ Similarly, a true bug species preferred preying on sedentary spider mites over a more mobile prey, likely due to the lack of effective defensive mechanisms in the sedentary prey.^28^ Active predatory odonate larvae consume more sedentary prey, as these prey are encountered more frequently.^29^ A simulation model examining the efficiency of active versus ambush predators in controlling prey populations found that active predators are particularly effective against sedentary prey.^30^ Lastly, the stomachs of anuran active predators predominantly consist of prey species with low mobility.^31^

In this study, I designed a spatially explicit individual‐based simulation model to explore the conditions under which active predators encounter sedentary prey more frequently than mobile prey, as predicted by previous studies on the foraging mode paradigm. I focus solely on encounter probabilities and do not assume differences in capture success between predators of different prey types. Successful capture depends on prey detection and prey subduing. Mobile prey may be more easily detected by predators than sedentary prey, while sedentary prey may be more easily subdued or less effective at evading attacks.^27^, ^29^, ^32^, ^33^, ^34^ To examine encounter probabilities, I modified the spatial pattern of sedentary prey and the search tactic of active predators. I also modified the movement directionality and speed of prey and active predators. Furthermore, ambush predators may compete with active predators for mobile prey, potentially affecting the encounter probabilities of the latter two. Lastly, I incorporated uncertain capture probability and prey reappearance after being captured.

## METHODS

**FIGURE 1:**
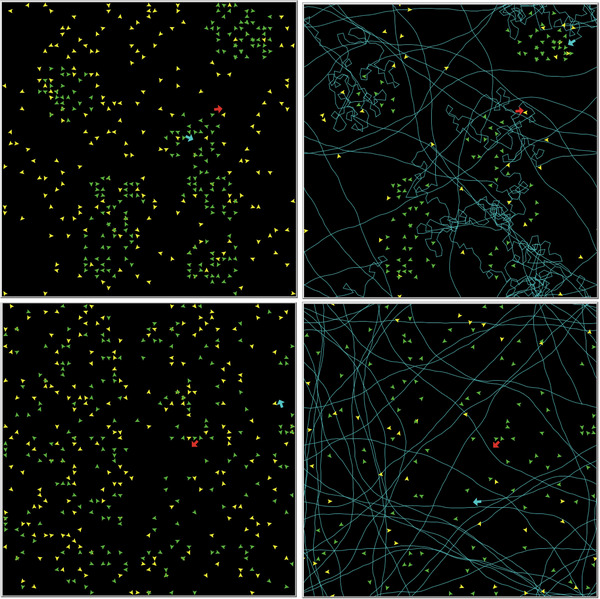
Four screenshots of the arena where the simulation takes place. Left: Initial setups of a clumped spatial pattern (top) and a random spatial pattern (bottom) of sedentary prey. Right: The final state of each setup, including the movement trajectories of the active predators (cyan lines). Note that in the clumped spatial pattern of sedentary prey, the active predator employs area‐restricted search, whereas in the random spatial pattern, it employs constant directional movement. Color legend: cyan = active predators, red = ambush predators, green = sedentary prey, yellow = mobile prey.

The simulation model is individual‐based and spatially explicit, developed in NetLogo 6.3.0.^35^, ^36^ It adheres to the key elements of the ODD protocol for simulation model description.^37^ The simulation flowchart is provided in Part S1 of the Supporting Information, and Figure 1 presents screenshots of the simulation's graphical presentation. The simulation is loosely based on a previous simulation addressing a different research question.^38^

### Purpose

The simulation aims to assess the relative encounter rates of an active predator with mobile and sedentary prey, both with identical initial densities. Several habitat, predator, and prey traits were manipulated to evaluate their effects on the relative encounter rates.

### Entities, state variables, and scales

#### Entities

The model includes four entities: (1) an active predator; (2) an ambush predator, present in some scenarios; (3) 200 mobile prey; and (4) 200 sedentary prey.

#### State variables

The state variables are the positions of predator/s and prey, movement directions and speed of active predators and mobile prey, and movement patterns (or the change in movement direction between two successive steps, resulting in varying degrees of directionality). Movement directionality level is a factor shown in previous simulation models to influence the relative success of active versus ambush predators.^38^, ^39^ The active predator can employ area‐restricted search (ARS) with less directional movement after encountering sedentary prey, or maintain a fixed movement directionality. ARS combines directional movement between prey patches and nondirectional movement within patches, optimizing the detection of clumped prey.^40^, ^41^, ^42^ Capture success of prey by predators can be certain (100%) or partial (50%). Capture upon encounter is often uncertain^43^, ^44^, ^45^ and depends on prey detection and subduing. Sedentary prey are typically well camouflaged, while mobile prey evade predators better.^46^, ^47^ For simplicity, I treat these components as equivalent in defining capture probability. The movement speed of the active predator and mobile prey is identical in most scenarios but can differ, with either moving twice the speed of the other. Movement speed has been shown in previous simulation models to affect encounter probabilities.^17^, ^39^ Lastly, I enabled prey reappearance, a natural process in many systems, such as wind dispersing or exposing new seeds for seed‐eaters.^48^, ^49^ If prey reappearance is enabled, there is a 10% probability per time step for a new mobile or sedentary prey to appear. New mobile prey start moving on the next time step, while new sedentary prey are positioned in a neighboring cell of one of the other sedentary prey.

#### Scales

The model comprises a two‐dimensional arena (51×51 cells). When active predators or mobile prey reach the arena boundaries, they re‐enter from the opposite side, maintaining their movement direction—a wrapped topology or torus model, similar to other individual‐based simulations.^50^, ^51^, ^52^ Further details on the impact of boundary‐handling methods and arena dimensions are provided in Parts S2 and S3 of the Supporting Information.

### Process overview and scheduling

Time is represented as discrete time steps. During each time step, mobile prey and active predators move a distance of one cell. While the arena is divided into discrete cells, predator and prey locations are continuous. Active predators and mobile prey exhibit correlated random walks, meaning movement direction in each time step depends on the previous step, introducing both a degree of persistence and stochasticity in movement directions. Mobile prey may employ either directional movement (turning up to 10° between time steps) or nondirectional movement (turning up to 120° between time steps). Active predators either use a fixed movement directionality, similar to mobile prey, or ARS, switching from directional movement (turns up to 10°) before encountering sedentary prey to nondirectional movement (turns of up to 120°) for 20 time steps after encountering sedentary prey. If another sedentary prey is encountered during this period, the 20–time‐step count resets. Prey capture occurs when a predator and prey occupy the same cell. Capture probability is typically 100% but is reduced to 50% in some treatments. If two prey items occupy the same cell as a predator, the predator captures both. If an active predator and an ambush predator occupy the same cell with a single prey, each has a 50% chance of capture. The simulation ends when the active predator captures more than 50% (200) of the total number of prey, mobile and sedentary combined. Capture is determined each time mobile prey or the active predator move, and once per time step for ambush predators. The sequence of the main events in each time step is as follows: moving mobile prey, checking for prey capture by predator, moving the active predator, checking for prey capture by predators, handling prey reappearance if enabled, and checking the stop condition.

### Design concepts

#### Basic principles

The simulation evaluates the relative encounter rates of active predators with mobile and sedentary prey under different conditions. The output is this relative encounter rate.

#### Interactions

The only interaction occurs when predators and prey share the same cell, resulting in prey capture and removal from the arena.

#### Stochasticity

Stochastic elements include the random initial positions of predators and prey, random components of movement direction for active predators and mobile prey, the probability for prey reappearance if enabled, and the reduced capture probability of prey if enabled.

### Initialization

The spatial distribution pattern of sedentary prey can be clumped or random. If it is clumped, eight random patch centers are chosen, and 25 sedentary prey items are distributed randomly within a 5‐cell radius of each center. If it is random, 200 sedentary prey items are distributed randomly in the arena. Next, 200 mobile prey items are randomly distributed in the arena. One active predator is placed at a random position, along with either one or zero ambush predators.

### Simulation scenarios

I examined eight treatments (summarized in Table 1): default conditions (a directionally moving active predator and mobile prey in a random spatial pattern of sedentary prey; and an active predator employing ARS in a clumped pattern of sedentary prey), mobile prey move nondirectionally, the active predator moves nondirectionally, both the active predator and mobile prey move nondirectionally, mobile prey move twice as fast as the active predator, the active predator moves twice as fast as mobile prey, uncertain prey capture (the probability is reduced to 50%), and prey reappearance (a probability of 10% of reappearance of a single prey item of each type per time step).

**TABLE 1:** Scenarios examined and the model parameters they modified.

Spat. Pat.	Mobile Prey Direc.	Active Pred. Direc.	Speed ×2	Capt. rate	Prey reappear?
Random	D	D	No	100%	No
Random	D	ARS	No	100%	No
Random	ND	D	No	100%	No
Random	D	ND	No	100%	No
Random	ND	ND	No	100%	No
Random	D	D	Prey	100%	No
Random	D	D	Pred.	100%	No
Random	D	D	No	50%	No
Random	D	D	No	100%	Yes
Clumped	D	D	No	100%	No
Clumped	D	ARS	No	100%	No
Clumped	ND	ARS	No	100%	No
Clumped	D	ND	No	100%	No
Clumped	ND	ND	No	100%	No
Clumped	D	ARS	Prey	100%	No
Clumped	D	ARS	Pred.	100%	No
Clumped	D	ARS	No	50%	No
Clumped	D	ARS	No	100%	Yes

*Note*: First (Random) and third (Clumped) scenarios examined. Ambush predators are absent in both, and scenarios 2 and 4 are identical to scenarios 1 and 3, respectively, except for one ambush predator present in each run.

Abbreviations: Amb. Pred. Direc., ambush predator directionality; ARS, area‐restricted search; Capt., capture; D, directionality; Mobile Prey Direc., mobile prey directionality; ND, nondirectional; Pred., predator.

These treatments were tested in four different scenarios: (1) a single active predator with a random spatial pattern of sedentary prey; (2) a single active predator with a clumped spatial pattern of sedentary prey; (3) a single active predator and a single ambush predator with a random spatial pattern of sedentary prey; and (4) a single active predator and a single ambush predator with a clumped spatial pattern of sedentary prey. When sedentary prey were clumped, the active predator employed ARS instead of fixed movement directionality. Consequently, the treatment involving nondirectionally moving prey was somewhat different based on the spatial pattern of sedentary prey (if it is clumped, active predators employ ARS, and if it is random, active predators use fixed directional movement). Part S4 of the Supporting Information presents the simulation durations for each scenario and treatment.

### Evaluations

I recorded the number of mobile prey and sedentary prey captured by the active predator and calculated the proportion of mobile prey captured out of the total prey captured by the active predator (hereafter, the proportion of mobile prey captured). Proportions greater than 0.5 indicate more frequent captures of mobile prey, whereas those smaller than 0.5 indicate more frequent captures of sedentary prey. Each scenario was replicated 50 times. I used bootstrapping to calculate the 95% confidence intervals (CIs; written in MATLAB (v. 2024b), The MathWorks Inc, Natick, Massachusetts). Treatments were considered different if their CIs did not overlap. The bootstrap procedure created 10,000 groups, each consisting of 50 replications, randomly selected with possible replacements. Each group was sorted in ascending order, and the CIs were determined as the values at the 0.025 × 10,000 and 0.0975 × 10,000 positions.

## RESULTS

### A single active predator, a random pattern of sedentary prey

**FIGURE 2:**
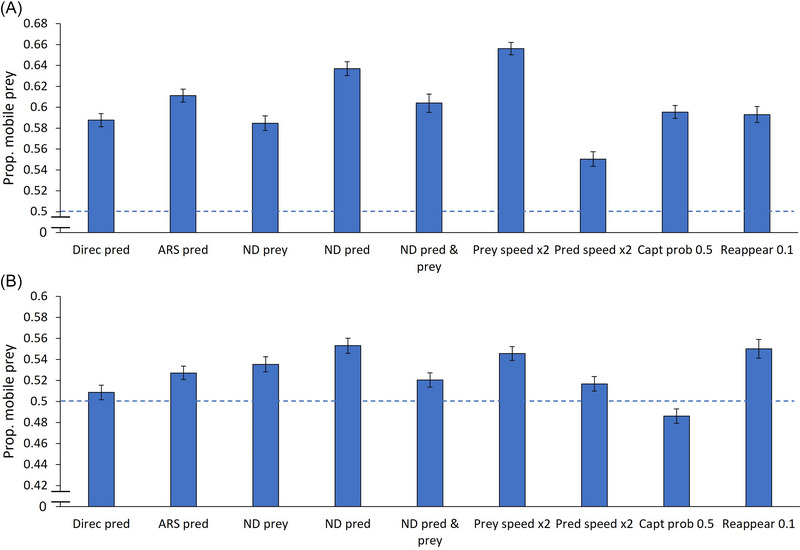
Proportions (means ± 95% CIs) of mobile prey captured by active predators when sedentary prey are distributed in a random spatial pattern under two conditions: (a) only active predators are present, and (b) both active and ambush predators are present. The segmented blue line crosses the *y*‐axis at 0.5, indicating no preference for either prey type. Note that the *y*‐axis does not start at zero. Abbreviations: ARS, area‐restricted search; capt prob, capture probability; Direc, directionally moving; ND, nondirectionally moving; pred, active predator; reappear, the probability for new prey to appear.

In the absence of ambush predators and with sedentary prey randomly distributed in space, active predators captured a higher proportion of mobile prey than sedentary prey across all treatments (0.588 under default conditions; results are summarized in Figure 2A). Two treatments greatly increased the proportions of mobile prey captured: prey moving at twice the speed of the active predator (0.656) and an active predator exhibiting nondirectional movement (0.637). Similarly, active predators employing ARS, characterized by nondirectional movement, at least partially, also encountered a higher proportion of mobile prey (0.611). In contrast, when the active predator moved at twice the speed of the mobile prey, the proportion of mobile prey captured dropped to the lowest observed value in this scenario (0.550). All other treatments produced results comparable to the default conditions.

### A single active predator and a single ambush predator, a random pattern of sedentary prey

Mobile prey were captured more frequently than sedentary prey in all treatments but one (results are summarized in Figure 2B). The exception was the uncertain prey capture treatment (0.486). The highest proportions of mobile prey captured occurred when the active predator moved nondirectionally (0.553), prey reappeared (0.550), and mobile prey moved twice as fast as the mobile predator (0.546). Nondirectional movement by mobile prey also resulted in a relatively high proportion of mobile prey captured (0.535).

### A single active predator, a clumped pattern of sedentary prey

**FIGURE 3:**
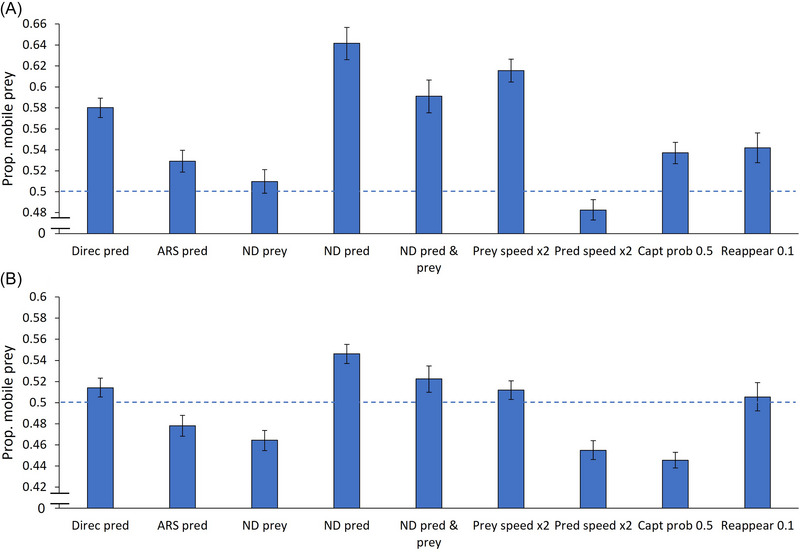
Proportions (means ± 95% CIs) of mobile prey captured by active predators when sedentary prey are distributed in a clumped spatial pattern under two conditions: (a) only active predators are present, and (b) both active and ambush predators are present. The segmented blue line crosses the *y*‐axis at 0.5, indicating no preference for either prey type. Note that the *y*‐axis does not start at zero. Abbreviations: ARS, area‐restricted search; capt prob, capture probability; Direc, directionally moving; ND, nondirectionally moving; pred, active predator; reappear, the probability for new prey to appear.

Mobile prey were captured more frequently than sedentary prey in most treatments (results are summarized in Figure 3A). The only exception occurred when the active predator moved twice as fast as the mobile prey (0.483). The second lowest proportion was observed when mobile prey moved nondirectionally (0.510). In contrast, three treatments yielded relatively high proportions of mobile prey captured: active predators moving nondirectionally (0.642), both the active predator and mobile prey move nondirectionally (0.591), and mobile prey moving twice as fast as the active predator (0.615).

### A single active predator and a single ambush predator, a clumped pattern of sedentary prey

Four treatments resulted in lower proportions of mobile prey captured compared to sedentary prey (results are summarized in Figure 3B). The two lowest proportions captured were observed under uncertain prey capture (0.445) and when the active predator moved twice as fast as the mobile prey (0.455). Both proportions were lower than the default treatment (0.478), where mobile prey were also less frequently captured than sedentary prey. Conversely, four treatments resulted in higher proportions of mobile prey captured. Two of these occurred when active predators moved with constant directionality level (and not using ARS), either directionally (0.514), nondirectionally (0.546), or when both active predators and prey move nondirectionally (0.522). When mobile prey moved twice as fast as the active predator, mobile prey were captured slightly more frequently (0.512). Finally, in the prey reappearance treatment, no clear priority was observed between mobile and sedentary prey (0.505).

## DISCUSSION

The simulation results varied depending on two primary factors: (1) the spatial distribution pattern of sedentary prey (clumped or random) and (2) the presence of ambush predators nearby. Ambush predators and a clumped pattern of sedentary prey reduced the proportion of mobile prey captured by active predators and promoted the specialization of active predators on sedentary prey. Although ambush predators had a greater influence than the spatial pattern of sedentary prey, their combined effect further decreased the proportion of mobile prey captured. Specific treatments interacted with these two factors to shape the outcomes. For example, when sedentary prey were randomly distributed, a shift in mobile prey movement from directional to nondirectional increased the proportion of mobile prey captured by active predators in the presence of ambush predators but had no effect in their absence. Similarly, prey reappearance influenced the proportion of mobile prey captured only in the presence of ambush predators, with no significant impact in their absence. Generally, the specialization of active predators on sedentary prey was quite weak with no more than 55.5% of sedentary prey captured by active predators of the total prey captured. This specialization is likely limited to specific conditions and is not as universal as previously suggested. The relative proportion of mobile prey captured by active predators thus depends on a complex interplay of multiple factors.

The presence of ambush predators has the greatest negative effect on the capture of mobile prey by active predators. The cooccurrence of active and ambush predators is common and may serve as a mechanism of character displacement and coexistence among predators.^53^, ^54^, ^55^, ^56^ In such cases, the simulation predicts prey specialization, at least to some extent. Specialization by active predators on sedentary prey also depends on the spatial distribution pattern of sedentary prey and the movement pattern of active predators. ARS increases the likelihood of encountering clumped sedentary prey, thereby reducing the proportion of mobile prey captured. The spatial pattern of prey may affect the predator's hunting success and movement patterns while searching.^57^, ^58^, ^59^ Prey spatial patterns can vary widely depending on numerous factors, including the scale of observation.^60^, ^61^

Several results were consistent across scenarios. First, active predators moving nondirectionally combined with mobile prey moving directionally resulted in high proportions of mobile prey captured in all scenarios. This occurred because active predators, covering space less efficiently and often re‐entering already searched areas, captured fewer sedentary prey. Second, mobile prey moving twice as fast as active predators increased the proportion of mobile prey captured across scenarios. Faster mobile prey cover a larger area, increasing their likelihood of encountering active predators. In turn, active predators cover a smaller area and the simulation ends before they encounter sufficient numbers of sedentary prey. Third, faster‐moving active predators captured lower proportions of mobile prey regardless of the spatial pattern of sedentary prey, but particularly in the absence of ambush predators. Competition for mobile prey imposed by ambush predators likely accelerates the depletion of mobile prey, reducing the advantage of the active predator's increased speed. Movement speed and directionality of both predators and prey have been previously shown to affect encounter rates.^17^, ^39^, ^62^, ^63^

Some patterns emerging from the simulation are scenario‐specific, like the effect of prey reappearance, which increases the proportion of mobile prey captured but only in the presence of ambush predators. This indicates that ambush predators deplete mobile prey as they compete with active predators for this prey type. When prey reappear, or when depletion is relaxed in other ways, the effect of reappearance is minor. On the other hand, when competition by ambush predators is strong (e.g., by increasing the number of prey items required to be captured by the active predator to end the simulation; Supporting Information, Part S5), the specialization of active predators on sedentary prey is strengthened. Similarly, uncertain prey capture decreases the proportion of prey captured but only in the presence of ambush predators. This is likely because the simulation runs longer in this scenario, giving ambush predators more time to capture mobile prey. In short, the results demonstrate how competition can lead to behavioral specialization: if a competitor makes a shared resource less accessible, a solution could be to focus on a less used resource or habitat.^64^, ^65^ Lastly, nondirectional prey movement had little effect except in scenarios where prey were randomly distributed and ambush predators were also present. In this case, low prey movement directionality increased the proportion of mobile prey captured. This is likely because mobile prey remained in the same area longer and were less likely to be captured by ambush predators. This effect was not observed in the absence of ambush predators and was weaker when active predators searched in a clumped spatial pattern of sedentary prey using ARS. In the latter case, the active predator's partially nondirectional movement reduced the impact of prey movement directionality.

Like all models, this model incorporates simplifying assumptions.^36^ To name a few, it assumes neither interference competition nor information sharing among predators, no interaction among prey, a homogenous test arena aside from the locations of predators and prey, no behavioral changes in predators or prey during the simulation, no movement costs for the active predators and mobile prey, and equal capture probabilities for mobile and sedentary prey. Relaxing these assumptions could alter the simulation outcome. For example, if predators were more efficient at capturing one prey type over the other, the proportion of prey capture would be heavily biased toward that type. Additionally, specific test conditions, such as the stopping criteria for each run, could influence the results (e.g., whether to stop after capturing more or fewer prey items; see Supporting Information, Part S5). Regarding potential future directions, it would be interesting to include in the simulation predator and prey birth and death processes, which may end up in either stable equilibrium conditions or cycles.

In conclusion, this study explored the conditions under which active predators are likely to specialize in sedentary prey, as suggested by foundational papers on the foraging mode paradigm. I found that active predators do not necessarily specialize in sedentary prey, and if there is a specialization, it is quite weak. However, two key scenarios promote such specialization: a clumped spatial pattern of sedentary prey, which is more efficiently discovered using ARS by the predators, and exploitation competition from co‐occurring ambush predators. Additional traits of prey or predators enabled such a specialization, such as faster movement by active predators (but only when the sedentary prey is clumped), and uncertain capture success upon encounter (but only in the presence of ambush predators). In contrast, prey reappearance increased the capture of mobile prey by active predators (but only in the presence of ambush predators). Faster movement of the mobile prey and nondirectional movement of the active predators consistently reduced the proportion of mobile prey captured across all scenarios. The interaction among these factors points to the complexity of predicting how specific conditions influence the relative discovery and capture of mobile versus sedentary prey. This highlights the importance of such spatial simulation models in formalizing predictions for experimental validation.

## COMPETING INTERESTS

I declare no competing interests.

## Supporting information



The dataset is provided as a separate Excel file.

The Supporting Information consists of five parts: (Part S1) The simulation's flow chart; (Part S2) modifying how to deal with the arena boundaries; (Part S3) modifying other selected parameters of the simulation by around 20%; (Part S4) the simulation durations per treatment per scenario; and (Part S5) modifying the simulation's stop condition.

## Data Availability

The dataset of this study is uploaded as an Excel file as a part of the Supporting Information.
