# Supplementary material for: Active predators do not necessarily specialize in sedentary prey: A simulation model
Source: Ann N Y Acad Sci. 2025 May 21;1549(1):199–207. doi: 10.1111/nyas.15379 (PMC12309432; doi:10.1111/nyas.15379)
Supplement: Supplementary file 2 — The Supporting Information consists of five parts: (Part S1) The simulation's flow chart; (Part S2) modifying how to deal with the arena boundaries; (Part S3) modifying other selected parameters of the simulation by around 20%; (Part S4) the simulation durations per treatment per scenario; and (Part S5) modifying the simulation's stop condition. [file NYAS-1549-199-s002.docx]

Supplementary Material for the Manuscript:

**Active predators do not necessarily specialize in sedentary prey: a simulation model**

By: Inon Scharf

The Supplementary Material contains five parts:

Part S1: The simulation’s flow chart.

Part S2: Modifying how to deal with the arena boundaries.

Part S3: Modifying selected parameters of the simulation by around 20%.

Part S4: Simulation durations per treatment per scenario.

Part S5: Modifying the simulation’s stop condition.

**Part S1: The simulation’s flow chart**


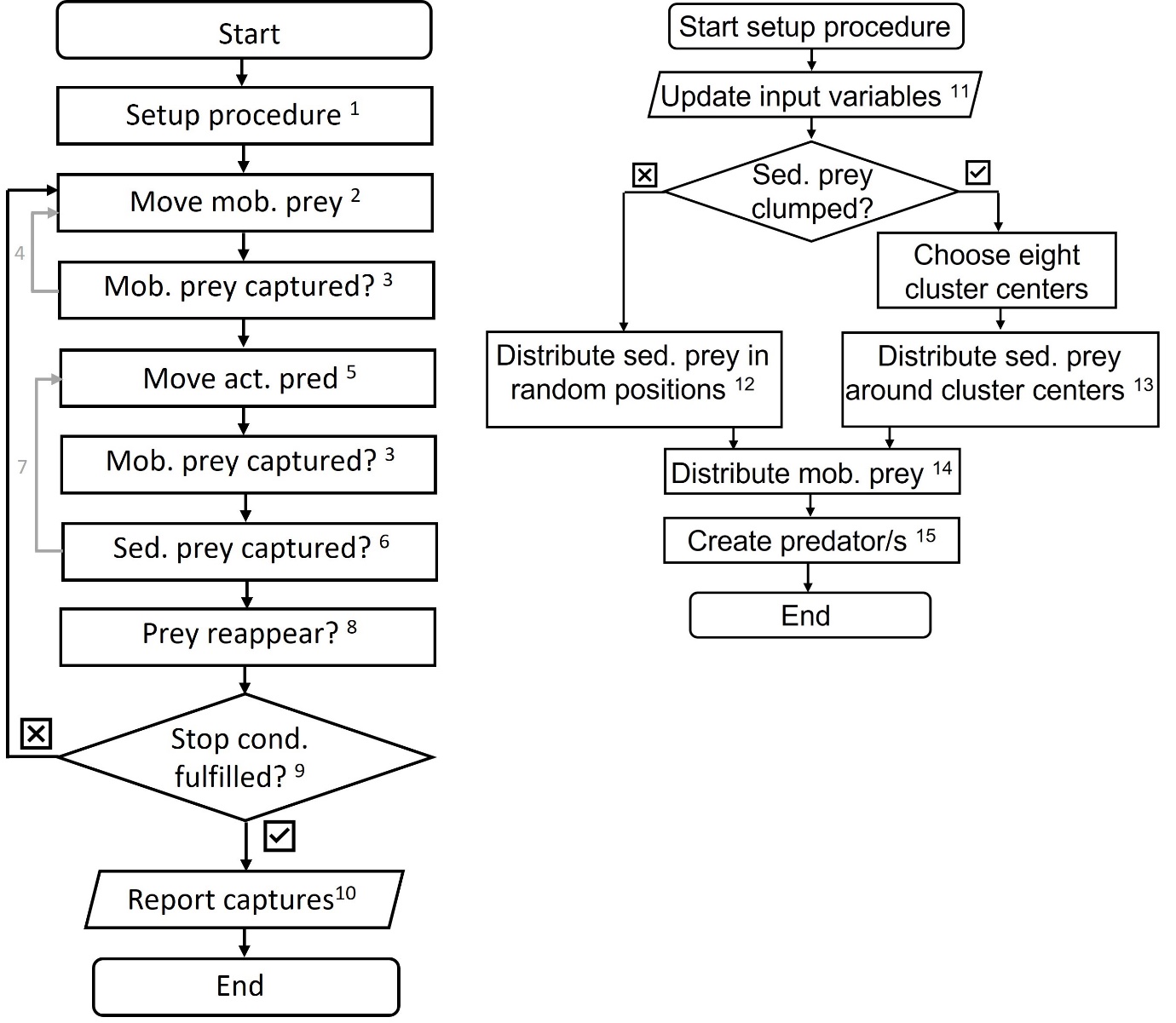


^1^ The setup procedure is displayed to the right.

^2^ Mobile prey move over one space unit. Following movement, prey can change their movement directionality.

^3^ If mobile prey and either an active or an ambush predator share the same cell, the prey is captured, it dies, and the number of mobile prey captured for the capturing predator is updated.

^4^ If mobile prey move twice as fast as the active predator, these two steps are repeated.

^5^ The active predator moves over one space unit. Following movement, the active predator can change its movement directionality. If ARS (area-restricted search) is applied, directionality changes during the 20-time steps after the capture of sedentary prey are more prominent.

^6^ If sedentary prey and either an active or an ambush predator share the same cell, the prey is captured, it dies, and the number of sedentary prey captured for the capturing predator is updated.

^7^ If the active predator moves twice as fast as the mobile prey, these three steps are repeated.

^8^ If prey reappearance is allowed, there is a 10% probability per time step for new prey per type to appear. A new mobile prey starts moving on the next time step. A sedentary prey is moved to one of the neighboring cells of existing sedentary prey.

^9^ If the stop condition is fulfilled, the simulation stops (the stop condition is 50% of the total prey items captured by the active predator).

^10^ The simulation output is the number of mobile and sedentary prey captured per predator.

^11^ The input variables are: the number of ambush predators (0 or 1), the spatial pattern of sedentary prey (random or clumped), whether the active predator uses ARS, the directionality level of the active predator and mobile prey, the probability of prey reappearance (0 or 0.1), the movement speed of the active predator and mobile prey (1 or 2), and the capture probability of mobile and sedentary prey (0.5 or 1).

^12^ Two hundred sedentary prey are placed in random positions.

^13^ Two hundred sedentary prey are placed around cluster centers, 25 per cluster.

^14^ Two hundred mobile prey are placed in random positions and are assigned a random movement direction.

^15^ An active predator is created, placed in a random position and allocated a random movement direction. An ambush predator, if included in the simulation, is placed in a random position.

**Part S2: Modifying how to deal with the arena boundaries**

In the manuscript, I applied a wrapped topology (or a “torus model”) according to which mobile prey or active predators that left the arena reentered it from the opposite side. To examine its effect on the simulation outcome, I used a non-wrapped topology as an alternative. Specifically, individuals reaching the arena boundaries could not leave and chose instead a new random direction. Topology had little effect on the simulation outcome. The only difference based on topology is when the active predators move non-directionally (fourth pairs of columns from the left), especially in the absence of ambush predators.


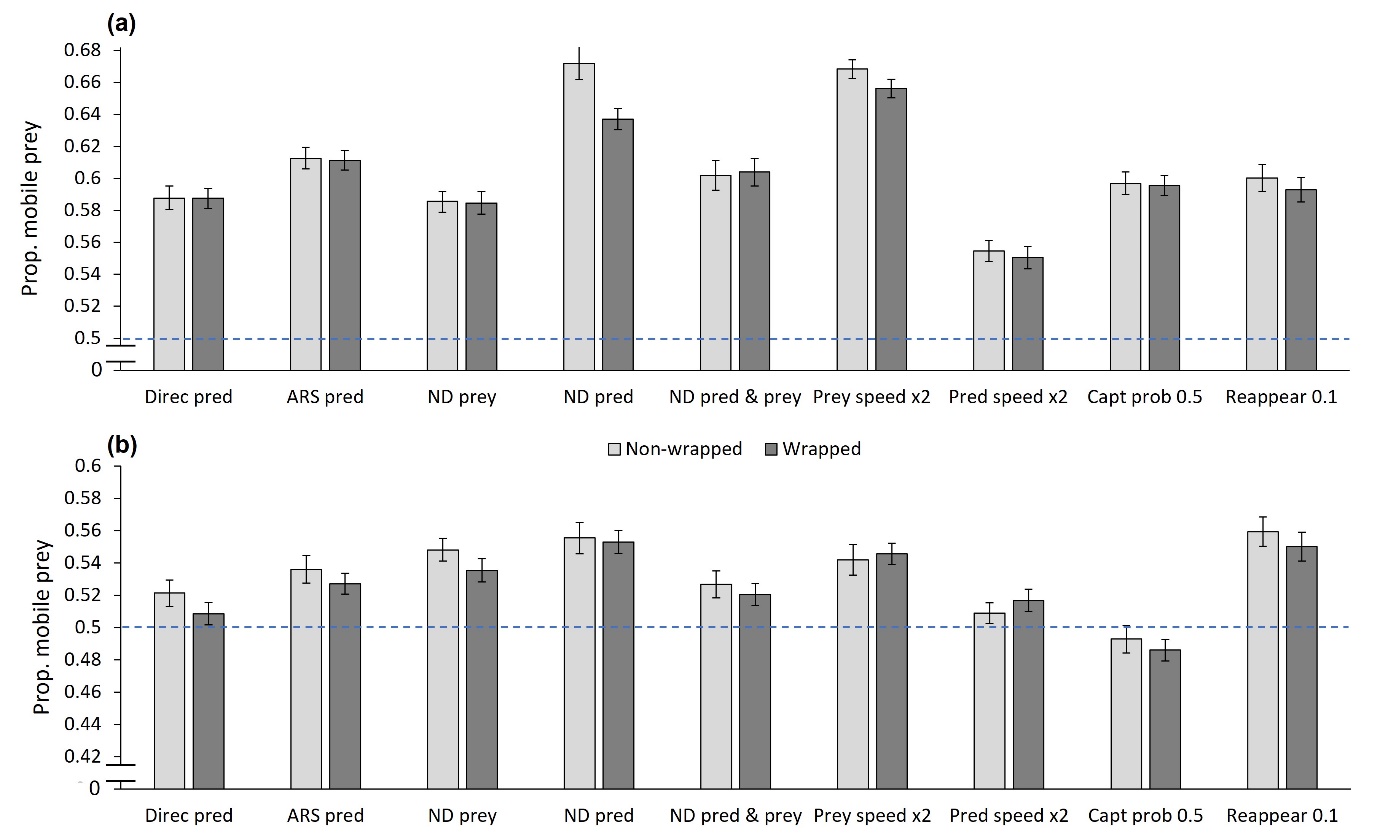


The proportions (means ± 95% CIs) of mobile prey caught by active predators (a) alone, and (b) in the presence of ambush predators. The sedentary prey is distributed in a random spatial pattern. Bright grey = non-wrapped topology, and dark grey = wrapped topology, taken from the manuscript. Direc = directionally moving, ARS = area-restricted search, ND = non-directionally moving, pred = active predator, capt prob = capture probability, reappear = the probability per time step for a new prey per prey type to appear. The segmented blue line crosses the y-axis at 0.5, indicating no preference for either prey type. Note that the y-axis does not start at zero.


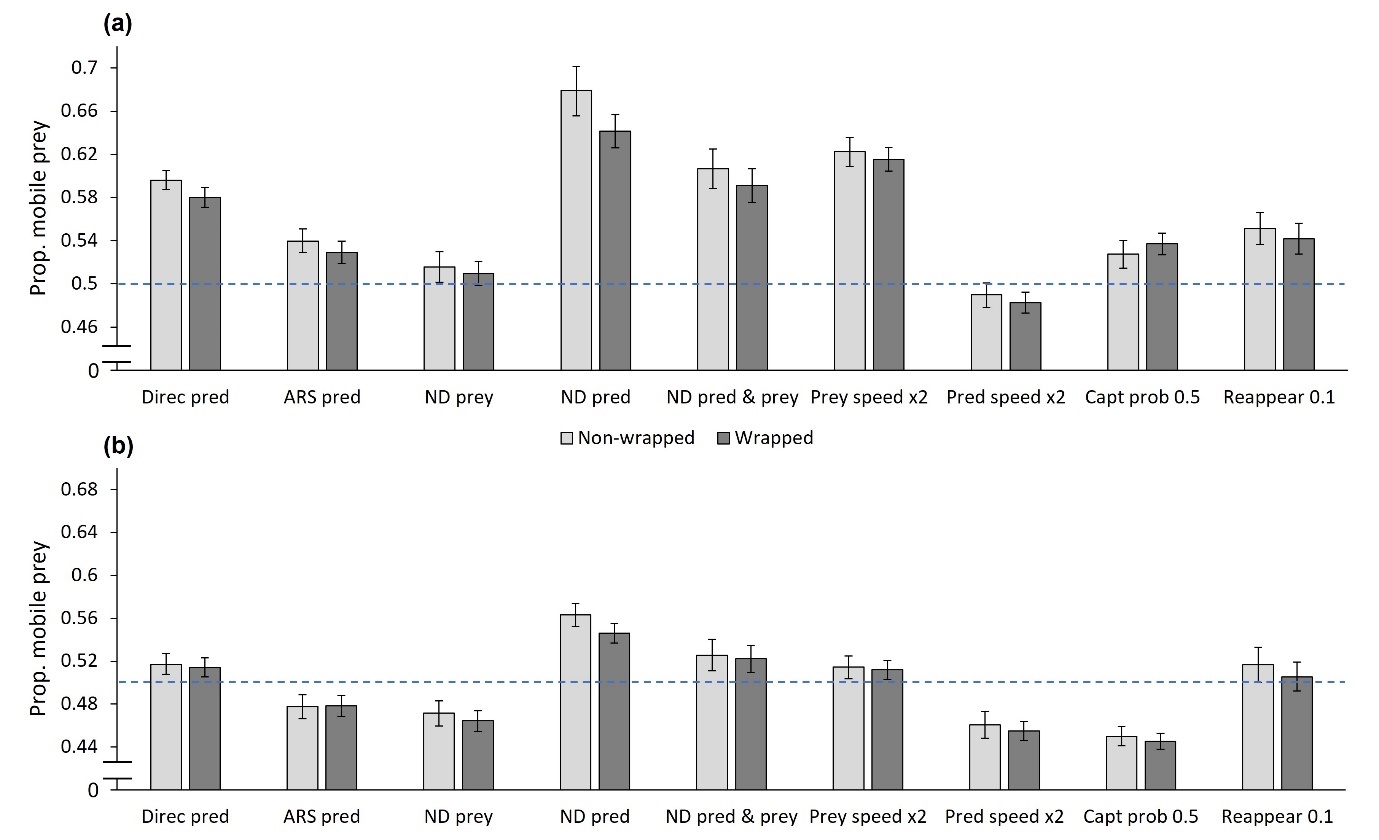


The proportions (means ± 95% CIs) of mobile prey caught by active predators (a) alone, and (b) in the presence of ambush predators. The sedentary prey is distributed in a clumped spatial pattern. Bright grey = non-wrapped topology, dark grey = wrapped topology, taken from the manuscript for comparison. Bright grey = non-wrapped topology, and dark grey = wrapped topology, as in the manuscript. Direc = directionally moving, ARS = area-restricted search, ND = non-directionally moving, pred = active predator, capt prob = capture probability, reappear = the probability per time step for a new prey per prey type to appear. The segmented blue line crosses the y-axis at 0.5, indicating no preference for either prey type. Note that the y-axis does not start at zero.

**Part S3: Modifying selected parameters of the simulation by ± around 20%**

a. Arena size:

The default arena size was 51 × 51 cells. I decreased and increased it to 46 × 46 and 56 × 56 cells. The effects on the proportions of mobile prey captured were minimal.


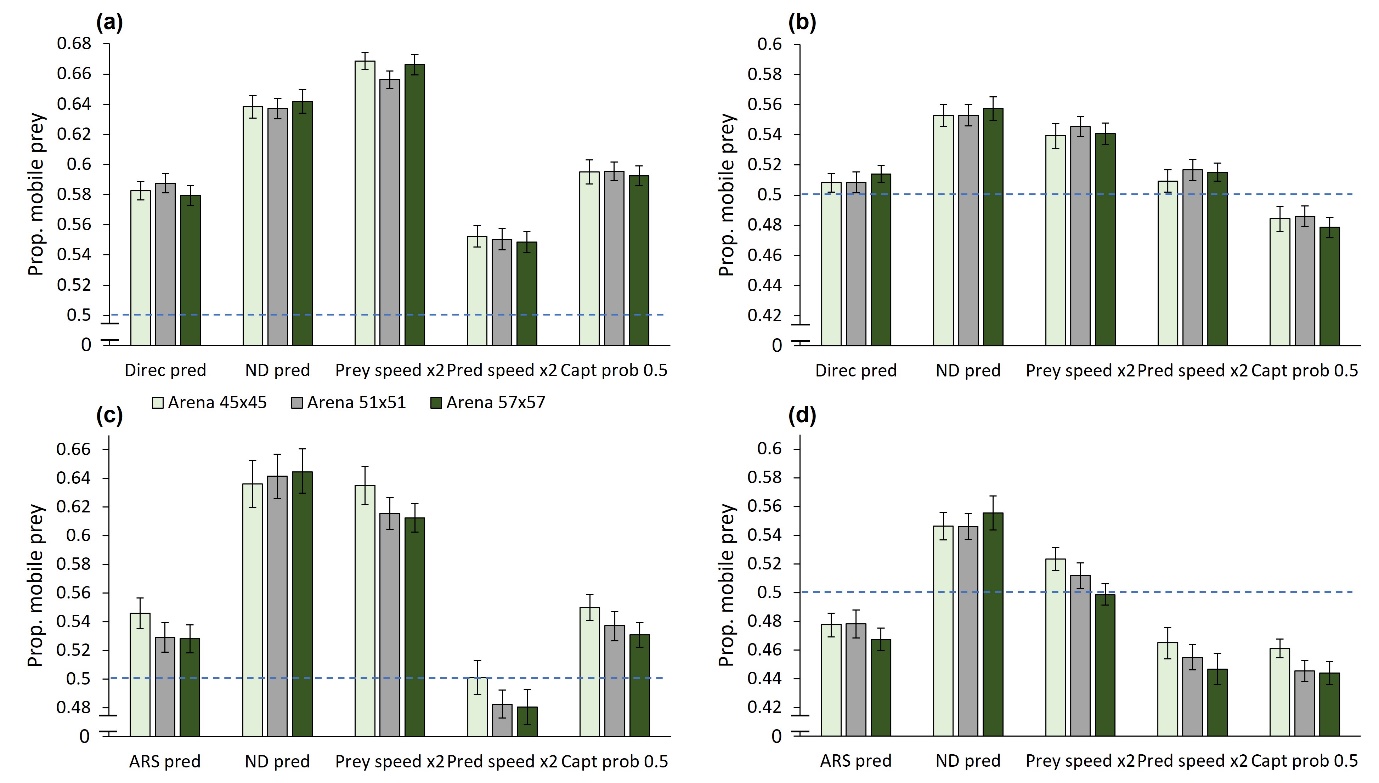


The proportions (means ± 95% CIs) of mobile prey captured by active predators when (a,c) only active predators are present, and (b,d) in the presence of both active and ambush predators; (a-b) a random spatial pattern of sedentary prey, and (c-d) a clumped spatial pattern of sedentary prey. Bright green, grey, and dark green stand for arenas of 45×45, 51×51, and 57×57 cells. Direc = directionally moving, ARS = area-restricted search, ND = non-directionally moving, pred = active predator, capt prob = capture probability. The segmented blue line crosses the y-axis at 0.5, indicating no preference for either prey type. Note that the y-axis does not start at zero.

b. The number of prey items:

The default number of prey items was 400, evenly split between mobile and sedentary ones. cells. I decreased the total number to 320 and increased it to 480. Note that the stop condition remained the same (50% of the total prey captured by active predators). The effects on the proportions of mobile prey captured were minimal.


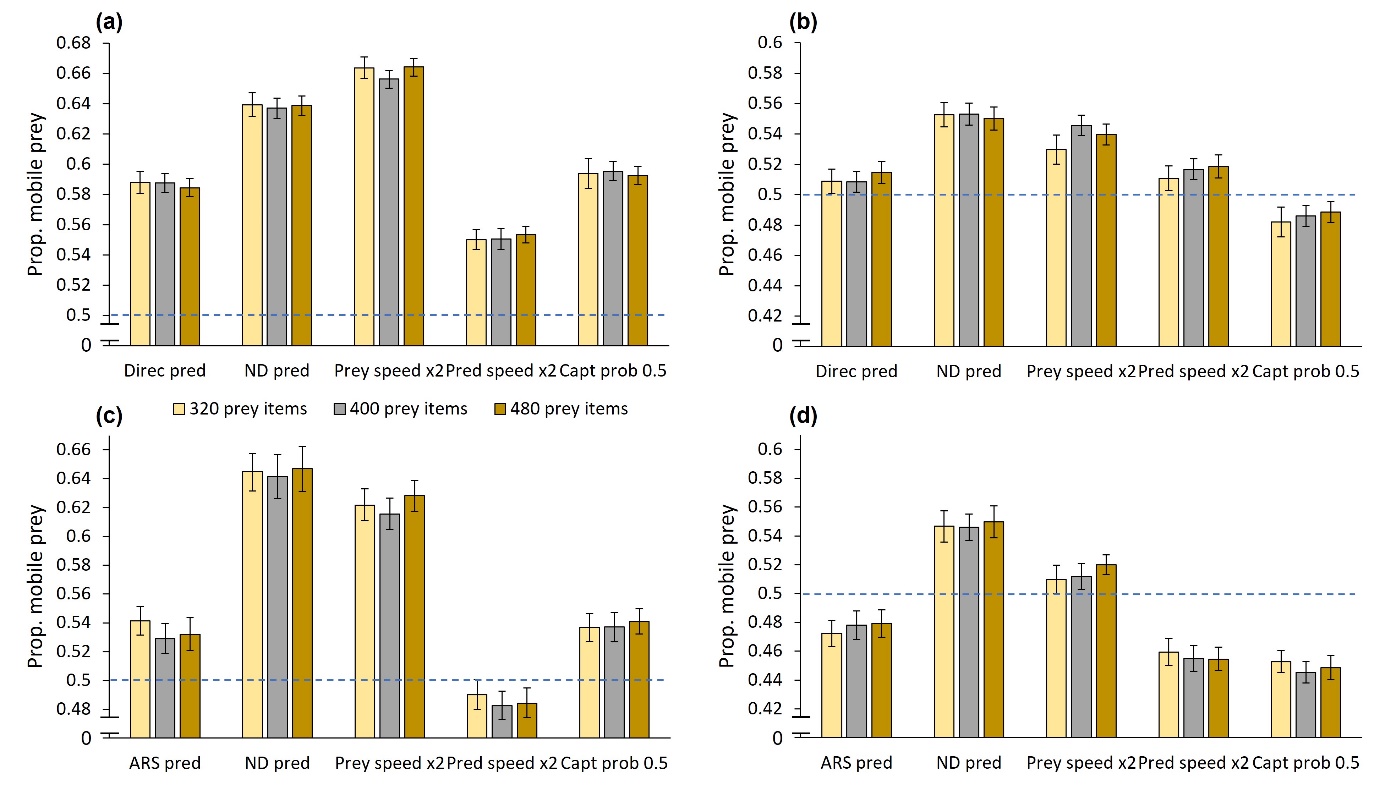


The proportions (means ± 95% CIs) of mobile prey captured by active predators when (a,c) only active predators are present, and (b,d) in the presence of both active and ambush predators; (a-b) a random spatial pattern of sedentary prey, and (c-d) a clumped spatial pattern of sedentary prey. Yellow, grey, and gold stand for initial numbers of 320, 400 (as in the manuscript), and 480 prey items. Direc = directionally moving, ARS = area-restricted search, ND = non-directionally moving, pred = active predator, capt prob = capture probability. The segmented blue line crosses the y-axis at 0.5, indicating no preference for either prey type. Note that the y-axis does not start at zero.

c. The duration of area-restricted search

The default duration of the non-directional movement employed by active predators following prey capture was 20-time steps. I decreased this number to 15 and increased it to 25. This was modified only in relevant scenarios and treatments (clumped spatial patterns of sedentary prey). The effect was minimal.


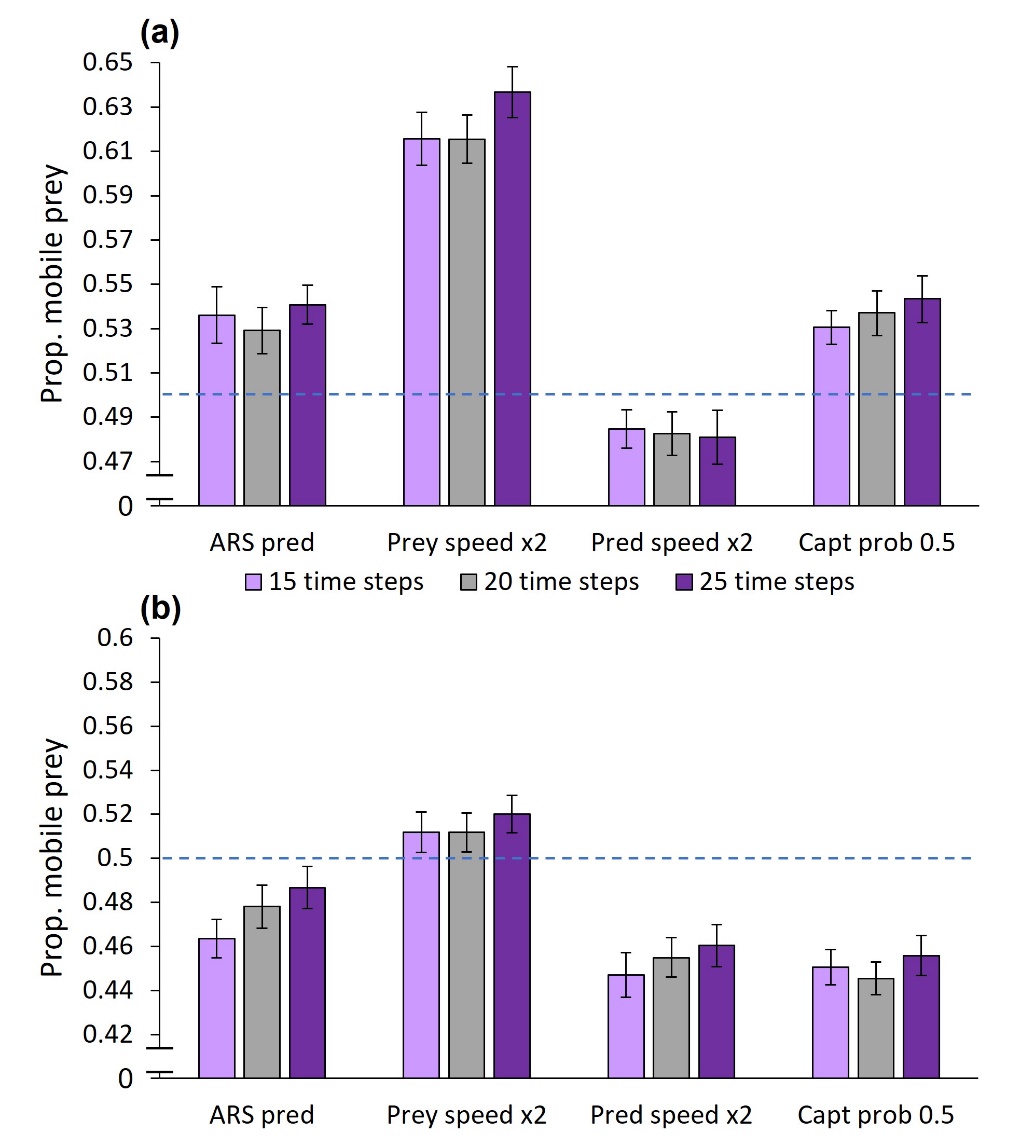


The proportions (means ± 95% CIs) of mobile prey captured by active predators when (a) only active predators are present, and (b) in the presence of both active and ambush predators. In all treatments, the sedentary prey was distributed in clumps. Bright purple, grey and dark purple stand for ARS durations of 15, 20, and 25 time steps. ARS = area-restricted search, pred = active predator, capt prob = capture probability. The segmented blue line crosses the y-axis at 0.5, indicating no preference for either prey type. Note that the y-axis does not start at zero.

**Part S4: Simulation durations per treatment per scenario**

The simulation was run for discrete time steps. The simulation duration is presented per scenario and treatment.


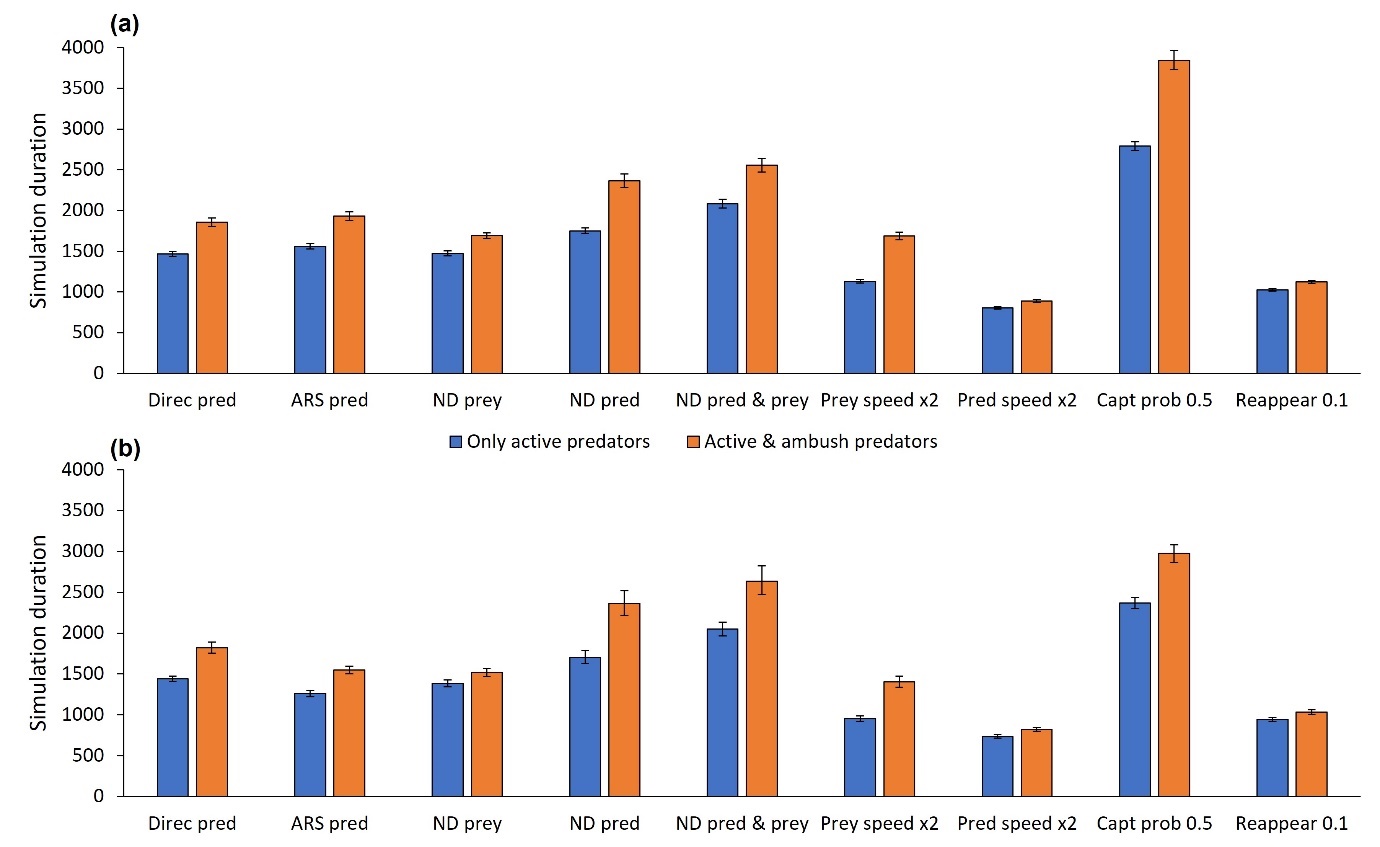


Simulation durations in time steps (means ± 95% CIs) per scenario and treatment: (a) random spatial pattern of sedentary prey, and (b) clumped spatial pattern of sedentary prey. Blue = only active predators are present, and orange = active and ambush predators are present. Direc = directionally moving, ARS = area-restricted search, ND = non-directionally moving, pred = active predator, capt prob = capture probability, reappear = the probability per time step for a new prey per prey type to appear.

**Part S5: Modifying the simulation’s stop condition**

The stop condition is the capture of 50% of all prey by the active predator (in the manuscript, 200 of 400 available prey items). I changed the stop condition to 25% or 75% of all prey (100 or 300). The later the simulation stops, the lower the proportion of mobile prey captured by active predators is, but with little interaction among treatments. This decrease is much more prominent in the presence of ambush predators than in their absence, leading to specialization of active predators on sedentary prey.


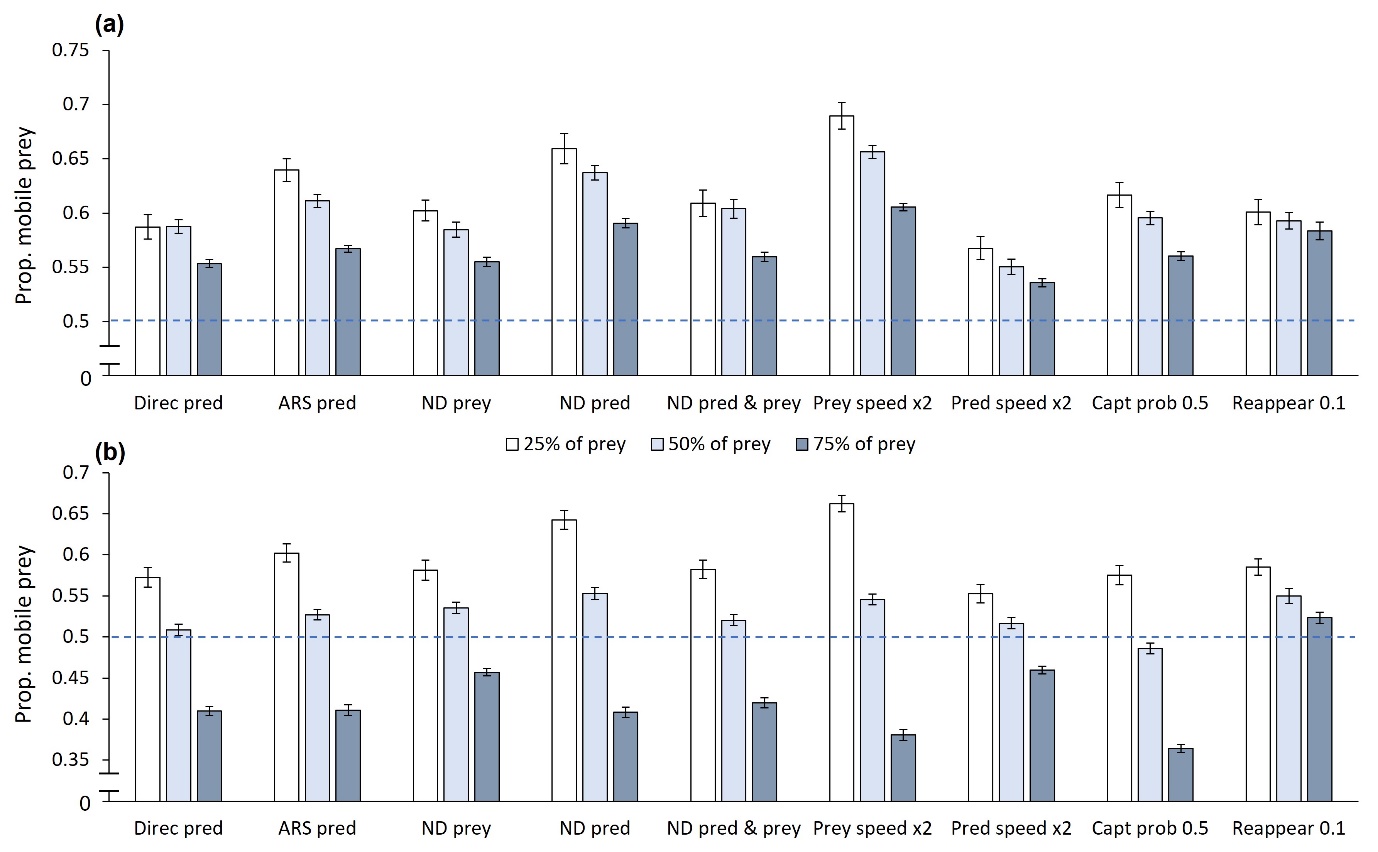


The proportions (means ± 95% CIs) of mobile prey caught by active predators (a) alone, and (b) in the presence of ambush predators. The sedentary prey is distributed in a random spatial pattern. White, bright blue, and dark blue = stop conditions of 25%, 50% (as in the manuscript), and 75% of the prey captured by the active predator. Direc = directionally moving, ARS = area-restricted search, ND = non-directionally moving, pred = active predator, capt prob = capture probability, reappear = the probability per time step for a new prey per prey type to appear. The segmented blue line crosses the y-axis at 0.5, indicating no preference for either prey type. Note that the y-axis does not start at zero.


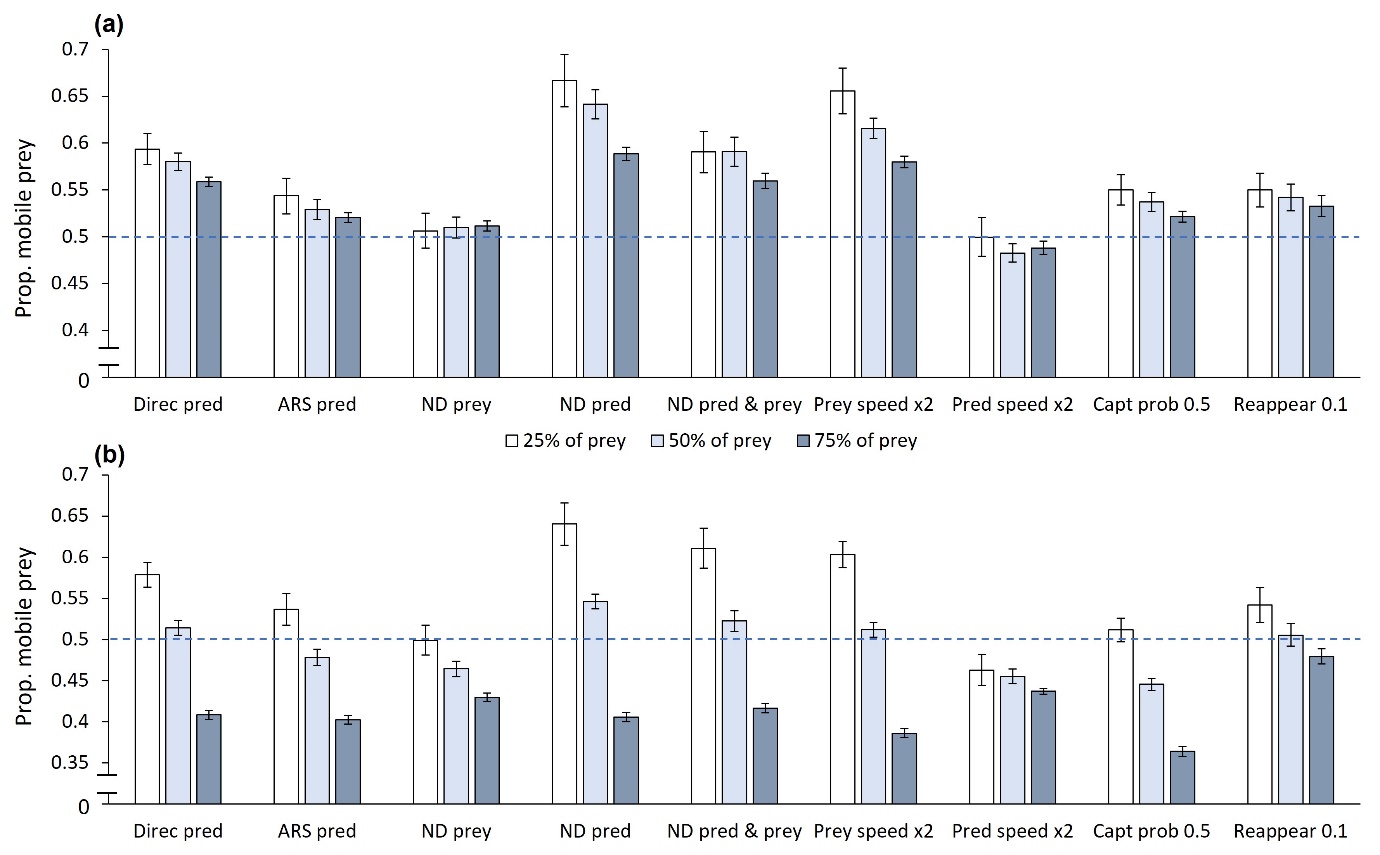


The proportions (means ± 95% CIs) of mobile prey caught by active predators (a) alone, and (b) in the presence of ambush predators. The sedentary prey is distributed in a clumped spatial pattern. White, bright blue, and dark blue = stop conditions of 25%, 50%, and 75% of the prey captured by the active predator. Direc = directionally moving, ARS = area-restricted search, ND = non-directionally moving, pred = active predator, capt prob = capture probability, reappear = the probability per time step for a new prey per prey type to appear. The segmented blue line crosses the y-axis at 0.5, indicating no preference for either prey type. Note that the y-axis does not start at zero.
